# FOXE1 regulates migration and invasion in thyroid cancer cells and targets ZEB1

**DOI:** 10.1530/ERC-19-0156

**Published:** 2019-12-16

**Authors:** Jesús Morillo-Bernal, Lara P Fernández, Pilar Santisteban

**Affiliations:** 1 Instituto de Investigaciones Biomédicas ‘Alberto Sols’, Consejo Superior Investigaciones Científicas, and Universidad Autónoma de Madrid (CSIC-UAM), Madrid, Spain; 2 Molecular Oncology Group, IMDEA Food Institute, CEI UAM-CSIC, Madrid, Spain; 3 Centro de Investigación Biomédica en Red de Cáncer (CIBERONC), Instituto de Salud Carlos III (ISCIII), Madrid, Spain

**Keywords:** thyroid cancer, FOXE1, ZEB1, SNP, EMT

## Abstract

FOXE1 is a thyroid-specific transcription factor essential for thyroid gland development and maintenance of the differentiated state. Interestingly, a strong association has been recently described between *FOXE1* expression and susceptibility to thyroid cancer, but little is known about the mechanisms underlying FOXE1-induced thyroid tumorigenesis. Here, we used a panel of human thyroid cancer-derived cell lines covering the spectrum of thyroid cancer phenotypes to examine *FOXE1* expression and to test for correlations between FOXE1 expression, the allele frequency of two SNPs and a length polymorphism in or near the FOXE1 locus associated with cancer susceptibility, and the migration ability of thyroid cancer cell lines. Results showed that FOXE1 expression correlated with differentiation status according to histological sub-type, but not with SNP genotype or cell migration ability. However, loss-and-gain-of-function experiments revealed that FOXE1 modulates cell migration, suggesting a role in epithelial-to-mesenchymal transition (EMT). Our previous genome-wide expression analysis identified *Zeb1*, a major EMT inducer, as a putative Foxe1 target gene. Indeed, gene silencing of* FOXE1* decreased *ZEB1* expression, whereas its overexpression increased *ZEB1* transcriptional activity. FOXE1 was found to directly interact with the *ZEB1* promoter. Lastly, *ZEB1* silencing decreased the ability of thyroid tumoral cells to migrate and invade, pointing to its importance in thyroid tumor mestastases. In conclusion, we have identified *ZEB1* as a *bona fide* target of FOXE1 in thyroid cancer cells, which provides new insights into the role of FOXE1 in regulating cell migration and invasion in thyroid cancer.

## Introduction

Thyroid cancer is the most commonly occurring endocrine malignancy and its incidence has steadily increased over the last four decades, accounting for 1% of all annual cancer diagnoses (Davies & Welch 2006, Lim *et al.* 2017). Papillary thyroid carcinoma (PTC), a carcinoma of follicular cell origin, is the most frequent form of differentiated thyroid carcinoma and represents 80–85% of all thyroid malignancies (Zaballos & Santisteban 2017).

Initiation and progression of thyroid cancer results from the acquisition of multiple genetic alterations. PTC is mostly driven by mutations that activate the MAPK (mitogen-activated protein kinase) signaling pathway (Zaballos & Santisteban 2017), which includes mutations in the intracellular transducer RAS and the serine/threonine kinase BRAF, and rearrangements in the cell membrane receptor tyrosine kinase RET (DeLellis 2006, Riesco-Eizaguirre & Santisteban 2016). Beyond these somatic alterations, PTC displays a strong hereditary component, since it shows the highest familial relative risk (8.60–10.30) in first-degree relatives of probands among cancers not displaying Mendelian inheritance (Goldgar *et al.* 1994, Pal *et al.* 2001).

Genome-wide association studies (GWAS) have identified SNPs associated with PTC risk (Gudmundsson *et al.* 2009, Matsuse *et al.* 2011, Mancikova *et al.* 2015). These allelic variations include rs965513, found in the proximal region of the *FOXE1* (Forkhead Box E1) gene (approximately 57 kb from the *FOXE1* locus) and rs1867277, within its promoter (NM_004473.3:c. −283G>A), and both are strongly associated with an increased risk of PTC (Landa *et al.* 2009, Gudmundsson *et al.* 2012, Jones *et al.* 2012).

FOXE1, formerly known as thyroid transcription factor-2, is located on chromosome 9q22 in humans and encodes a DNA-binding protein belonging to the forkhead/winged-helix family, a superfamily of evolutionarily conserved transcriptional regulators that share a highly conserved forkhead box or winged helix DNA-binding domain (Chadwick *et al.* 1997, Cuesta *et al.* 2007). This transcription factor possesses a polymorphic polyalanine (poly-A) tract just distal to its DNA-binding domain (rs71369530), which varies between 11 and 22 alanine residues, although FOXE1^14Ala^ and FOXE1^16Ala^ account for greater than 98% of reported alleles (Macchia *et al.* 1999, Kallel *et al.* 2010).

*FOXE1* is a thyroid-specific transcription factor that, together with PAX8 and NKX2-1, coordinately maintains the differentiated state of the thyroid gland and is also essential for its correct development (Zannini *et al.* 1997, Fernandez *et al.* 2015). Foxe1 is also a key player in thyroid organogenesis, as its expression during early thyroid development is required for thyrocyte precursor migration (De Felice *et al.* 1998, De Felice & Di Lauro 2004, Parlato *et al.* 2004, Fernandez *et al.* 2015). In the differentiated thyroid, Foxe1 is a transcriptional activator of the thyroperoxidase and thyroglobulin genes and mediates the ability of cells to respond to external stimuli including thyroid stimulating hormone, insulin-like growth factor-1, and transforming growth factor-β (Santisteban *et al.* 1992, Ortiz *et al.* 1999, Lopez-Marquez *et al.* 2019). A previous genomic study by our group in a rat thyroid follicular cell line identified two thyroid-specific genes (*Duox2* and *Slc5a5*) among other genes (including *Cdh1* and *Nr4a2*) as novel Foxe1 targets (Fernandez *et al.* 2013).

Increasing evidence from genetic studies associates *FOXE1* with PTC, implicating it as a susceptibility gene in thyroid cancer; however, its involvement in the initiation and progression of these tumors is unknown. Likewise, several studies have reported the likely contribution of *FOXE1* to carcinogenesis in breast cancer (Park *et al.* 2012), pancreatic cancer (Sato *et al.* 2003), and basal and squamous cell carcinomas of the skin (Eichberger *et al.* 2004, Venza *et al.* 2010). In the context of thyroid carcinoma, several studies have focused on relating FOXE1 expression and localization in cancer cells to tumor development. For instance, FOXE1 overexpression has been described in PTC (Nonaka *et al.* 2008, Bychkov *et al.* 2013), as well as a gradual decrease in its nuclear expression according to the degree of tumor dedifferentiation, along with cytoplasmic accumulation (Zhang *et al.* 2006). This abnormal localization of FOXE1 could be related to thyroid tumorigenesis. In addition, an association has been described between the *FOXE1* poly-A repeat region and PTC (Bullock *et al.* 2012).

These observations, together with the genetic studies associating SNPs in and near the *FOXE1* locus with thyroid cancer risk, and the altered expression of FOXE1 (Landa *et al.* 2009, He *et al.* 2015*a*, Wang *et al.* 2017), have motivated us to further investigate the role of FOXE1 in thyroid cancer, to try to better understand the dual role of this transcription factor as both a differentiation and a tumoral factor. Accordingly, in the present study we analyzed FOXE1 expression levels in different thyroid cancer cell lines and looked for potential correlations with the genotypes of SNPs rs965513 and rs1867277 and with the length polymorphism rs71369530. Furthermore, we explored FOXE1-mediated regulation of epithelial-to-mesenchymal transition (EMT) by analyzing the expression of new genes regulated by FOXE1 in thyroid cells. Among them, *ZEB1*, a major EMT inducer, emerged as a putative FOXE1 target gene. Lastly, we studied the mechanism by which FOXE1 controls ZEB1 in thyroid cancer cell lines and demonstrated the involvement of ZEB1 in the regulation of EMT in thyroid cancer cells.

## Methods and materials

### Cell culture

Human thyroid cancer cell lines were obtained from the following sources: BCPAP and NIM from Dr M Santoro (University of Federico II, Naples, Italy); C643, Hth7, Hth83, and SW1736 from Dr N E Heldin (University of Uppsala, Uppsala, Sweden); FTC-133, K1 and Nthy-Ori-3.1 from the European Collection of Authenticated Cell Cultures (ECACC; Salisbury, Wiltshire, UK); WRO-82-1 from Dr G J F Juillard (University of California-Los Angeles School of Medicine, Los Angeles, CA, USA); TPC1 from Dr A P Dackiw (Johns Hopkins University, Baltimore, MD, USA); KTC-1 and KTC-2 from Dr Junichi Kurebayashi (Kawasaki Medical School, Japan); Cal62, ML-1, TT206-C09, and 8505c from the Leibniz-Institut DSMZ-German Collection of Microorganisms and Cell Cultures (Braunschweig, Germany); T235 and T238 from Dr Lucia Roque (Portuguese Cancer Institute, Lisbon, Portugal); and OCUT2 from Dr James A Fagin (Memorial Sloan Kettering Cancer Center, New York, NY, USA). All thyroid cancer cell lines and HeLa cells were grown in Dulbecco’s modified Eagle’s medium (DMEM). The human thyroid cell line Nthy-Ori-3.1 (ECACC #90011609) was grown in Roswell Park Memorial Institue 1640 medium. All growth media were supplemented with 10% fetal bovine serum (FBS), 50 U/mL penicillin, 50 μg/mL streptomycin, and 2 mmol/l-glutamine.

PCCl3 thyroid follicular cells, a continuous rat differentiated cell line, were cultured in Coon’s modified Ham’s F-12 medium supplemented with 5% donor calf serum (Thermo Fisher Scientific), and a six-hormone medium mixture: 1 nmol/L bovine thyroid-stimulating hormone, 10 μg/mL insulin, 10 ng/mL somatostatin, 5 μg/mL transferrin, 10 nmol/L hydrocortisone, and 10 ng/mL glycyl-l-histidyl-l-lysine acetate; all from Sigma-Aldrich.

All cell lines were used up to ten passages, maintained in 5% v/v CO_2_ at 37°C, and authenticated every 6 months by short tandem repeat profiling using the Applied Biosystems Identifier kit, at the Genomic Facility, Institute of Biomedical Research (IIBm; Madrid, Spain).

### RT-qPCR

RNA was extracted with TRIzol (Thermo Fisher Scientific), and 1 µg was added to a reverse-transcriptase reaction mix (M-MLV; Promega Co.,). Quantitative PCR (qPCR) was conducted on the Mx3000P QPCR platform (Agilent Technologies). Reactions were performed in triplicate with the indicated primers and templates using the KAPA SYBR FAST qPCR Master Mix (Merck KGaA) for 40 cycles. Relative gene expression levels were quantified using the comparative threshold cycle 2^−ΔΔCt^ method (Livak & Schmittgen 2001) by normalizing transcript levels to the expression of a housekeeping gene. The sequences of the specific primers purchased from Sigma-Aldrich are listed in Supplementary Table 1 (see section on supplementary materials given at the end of this article).

### Western blotting

Total protein extracts were obtained by scraping cells in RIPA buffer containing a protease inhibitor cocktail (Roche). Equal amounts of protein (30 µg) were separated by SDS-PAGE, transferred to nitrocellulose membranes, blocked, and incubated overnight with primary antibodies diluted in phosphate buffered saline (PBS) 0.1% v/v Tween 20 containing 5% w/v nonfat dry milk. Horseradish peroxidase (HRP)-conjugated secondary antibodies were incubated for 1 h at room temperature and binding was detected using enhanced chemiluminescence reagents (Thermo Fisher Scientific). The following antibodies were used in this study: anti-rat Foxe1 (#PA0200, Biopat Milan, Italy); anti-human FOXE1 (#ab5080, Abcam); anti-E-cadherin (#BD 610182; BD Biosciences, Bedford, MA); anti-ZEB1 (D80D3 #3396; Cell Signaling Technology), and anti-β-actin (#sc-1616; Santa Cruz Biotechnology).

### SNP genotyping

Genomic DNA from thyroid cell lines was extracted using the traditional saline method. DNA regions containing the two thyroid cancer-associated SNPs and the poly-A tract were amplified by PCR using specific primer pairs (Supplementary Table 2). PCR amplification was performed using KAPA Taq DNA polymerase (Merck KGaA) in a total volume of 15 μL containing 50 ng of DNA. PCR products were purified using the PureLink PCR Purification Kit (Invitrogen). Sequence analysis was performed on the ABI 3700 automated DNA sequencer (Applied Biosystems) using BigDye Terminator chemistry.

### RNA interference, plasmids, and transfection

For *FOXE1* gene silencing studies, cells were transfected with 25 nmol/L of FOXE1 siRNA (RatFoxe1 ON-TARGET Plus SMART pool or HumanFOXE1 ON-TARGET Plus SMART pool) or with scrambled siRNA (ON-TARGET Plus Non-targeting pool) using Dharma-FECT 1 Transfection Reagent (Dharmacon). For *ZEB1* gene silencing studies, human ZEB1 silencer® select siRNAs n269441 (siZEB1-1) and n269443 (siZEB1-2) were used (Thermo Fisher Scientific) following the same procedure as described previously. For *FOXE1* overexpressing studies, 1.5 µg of human *FOXE*-Flag or empty-Flag expression vectors (Clifton-Bligh* et al.* 1998, Carre* et al.* 2007) were transiently transfected. The day before transfection, cells were seeded in a six-well culture plate at a density of 2 × 10^5^ cells per well. Samples were harvested in duplicate at different time points (24, 48, and 72 h) after transfection, and total RNA and protein was extracted.

### Chromatin immunoprecipitation and electrophoretic mobility shift assay

Chromatin immunoprecipitation (ChIP) was performed as previously described (Fernandez *et al.* 2013) using the HighCell ChIP Kit (Diagenode Inc., Denville, NJ, USA). Cross-linked PCCl3 chromatin was immunoprecipitated using a polyclonal antibody against Foxe1 (Biopat, Milan, Italy). Two independent ChIP experiments were carried out using two different batches of Foxe1 antibody. Immunoprecipitated Foxe1 and input samples were assayed by qPCR using specific primers for the analyzed region on the *Zeb1* promoter (Supplementary Table 3). The known Foxe1 target *Tpo* was used as a positive control for immunoprecipitation, whereas two regions located −2 kb (*cis* 2 kb) and −2.5 kb (*cis* 2.5 kb) upstream of the transcription start site were used as negative controls. PCR reactions were performed in triplicate using the SYBR Green PCR Kit (Kapa Biosystems, Woburn, MA, USA). The enrichment of target sequences in ChIP experiments was calculated relative to the negative controls and normalized to their relative amplification in the input sample (Ruiz-Llorente *et al.* 2012).

Electrophoretic mobility shift assays were performed using an oligonucleotide probe derived from the *in silico*-identified FOXE1-binding site within the human *ZEB1* promoter (Oligo FOXE1: 5′-ATTCAAATAAACACTTGCATTTTA-3′). As a control, the Foxe1-binding site oligonucleotide derived from the rat *Tpo* promoter was used (Aza-Blanc *et al.* 1993). Probes were labeled with [γ^32^P]-ATP using T4 polynucleotide kinase (Promega) and purified using Quick Spin G-25 Sephadex columns (Roche Life Sciences). Recombinant FOXE1 was produced by *in vitro* transcription-translation using the TNT-coupled reticulocyte lysate system (Promega) and incubated with the labeled probe. Binding reactions were performed in a buffer containing 40 mmol/L Hepes, pH 7.9, 75 mmol/L KCl, 0.2 mmol/L EDTA, 0.5 mmol/L dithiothreitol, 150 ng/μL poly(dI-dC), and 5% w/v Ficoll at room temperature for 30 min. Samples were electrophoresed on a 5% w/v polyacrylamide gel in 0.5× Tris borate-EDTA. For competition, a 100-fold excess of the same (‘related’) or different (‘unrelated’) unlabeled oligonucleotides were used, as indicated in each experiment.

### Luciferase assay

HeLa cells were seeded at a density of 2 × 10^5^ cells per well in six-well tissue culture plates 24 h before transfection. Transfections were performed using the calcium phosphate co-precipitation method (Chen & Okayama 1988). The human *ZEB1* gene promoter (Dave *et al.* 2011) was transiently transfected alone (1.5 µg) or in combination with 0.5 µg of a human *FOXE*-Flag (Clifton-Bligh *et al.* 1998, Carre *et al.* 2007) expression vector. One hundred nanograms of the CMV *Renilla* vector were cotransfected to assess transfection efficiency. After 48 h, cells were harvested, lysed, and analyzed for Luciferase and *Renilla* activities using the Dual-Luciferase Reporter Assay System (Promega).

### Migration assay

Cell migration was evaluated using scratch wound healing assays. Cells were seeded on a six-well plate and allowed to reach confluence. Twenty-four hours after transfection, cells were treated for 2 h with 10 µg/mL mitomycin C in medium with 10% FBS to inhibit proliferation. After treatment, a single wound in the center of cell monolayer was made with a 10 μL pipette tip and cell debris was removed by washing with PBS. After 4, 8, 24, and 48 h of incubation in serum-free medium, the wound closure areas were visualized under an inverted microscope and imaged. Each experiment was performed in triplicate.

### Invasion assay

Cell invasion/migration was analyzed in Transwell assays using BD BioCoat Matrigel Invasion Chambers (BD Biosciences). In total, 2 × 10^5^ cells were suspended in 500 µL of serum-free medium and seeded into the upper chamber. The lower chamber of the Transwell was filled with 500 µL DMEM containing 20% FBS as a chemoattractant. After 18 h of incubation, cells on the surface of upper chamber were removed by scraping with a cotton swab. The invaded/migrated cells on the lower surface of the filter were fixed with 4% v/v paraformaldehyde, stained with 0.1% w/v crystal violet, imaged, and quantified by counting cells in five random fields. Experiments were performed three times in triplicate.

### Bioinformatics predictions

The TCGA database was queried to assess the correlations between mRNA levels and clinical features. Firebrowse (http://firebrowse.org) was used to analyze FOXE1 mRNA levels in different tumor types. The cBioPortal (http://www.cbioportal.org) and the Cancer Regulome Explorer (http://explorer.cancerregulome.org) data portals were used to obtain the correlations using the thyroid carcinoma dataset (THCA).

Microarray data of FOXE1 levels in anaplastic thyroid carcinomas (ATCs) comparing with normal thyroid tissues were searched in The Gene Expression Omnibus (GEO) database. Two microarray datasets GSE33630 (Tomas *et al.* 2012) and GSE65144 (von Roemeling *et al.* 2015) were analyzed.

### Statistical analysis

All data are reported as mean ± s.e.m. or mean ± s.d. Comparisons between two groups were made using two-tailed Student’s unpaired *t*-test. Statistical analysis was performed with GraphPad Prism software (GraphPad Software Inc.). Differences were considered statistically significant at *P* < 0.05. Associations between TCGA data and differentiation score and between FOXE1 expression and migration were assessed using Pearson’s (*r*) test.

## Results

### FOXE1 expression levels correlate with differentiation status in human thyroid cancer cell lines

**Figure 1:**
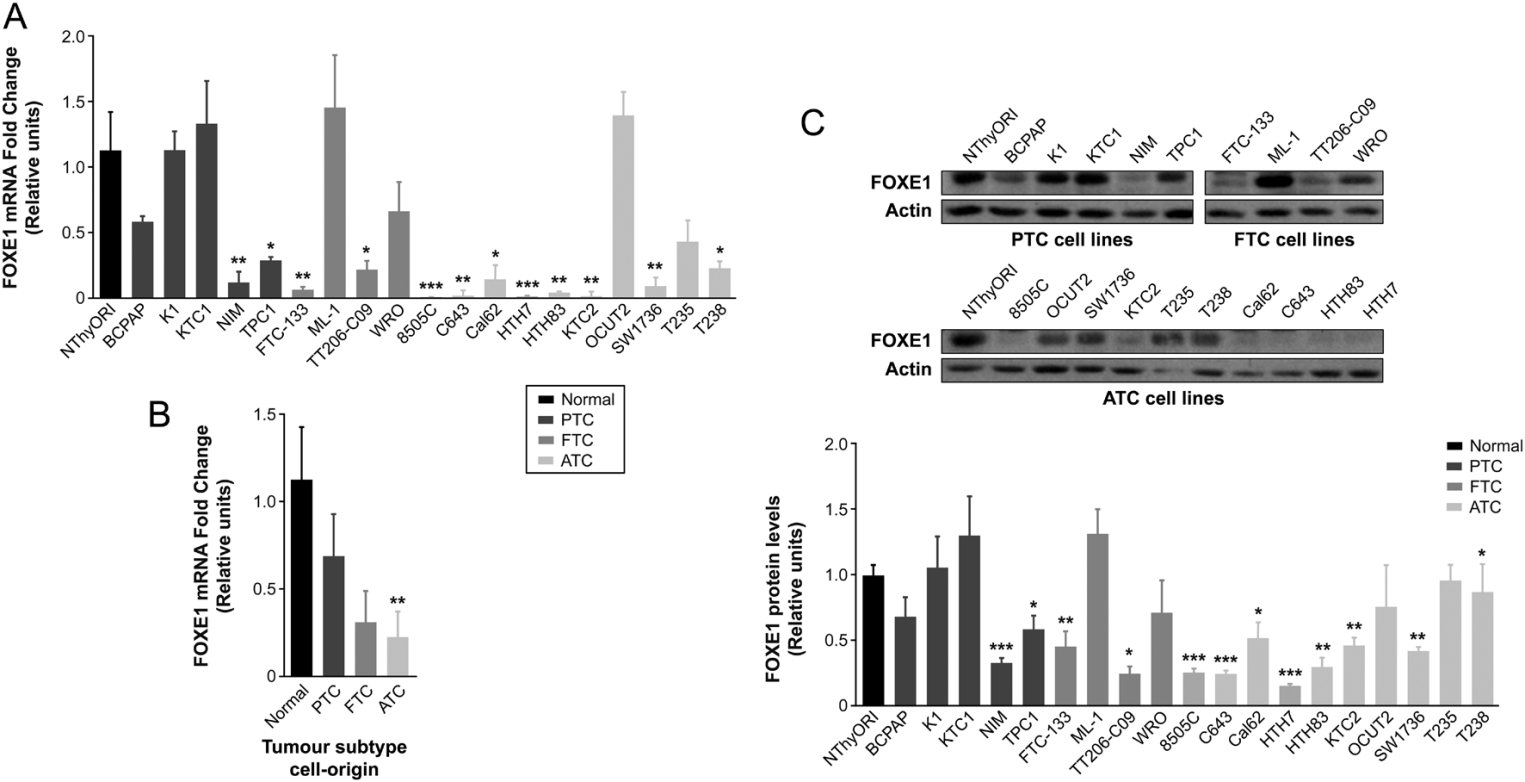
FOXE1 expression in thyroid cancer cell lines. (A) *FOXE1* relative mRNA expression in thyroid cell lines. Gene expression was normalized to expression of *GAPDH*. Values represent mean ± s.e.m. of three independent experiments. *P < 0.05, **P < 0.01, and ***P < 0.001 *vs* NThyORI control cells. (B) *FOXE1* relative mRNA expression according to tumor subtype-cell origin. Values represent mean ± s.e.m. **P < 0.01 *vs* NThyORI control cells. (C) Upper panel: Representative Western blot of three independent experiments showing FOXE1 protein levels in thyroid cell lines. Actin was used as a loading control. Lower panel: FOXE1 protein expression in thyroid cell lines normalized to expression of actin. Values represent mean ± s.e.m. of three independent experiments. *P < 0.05, **P < 0.001, and ***P < 0.001 *vs* NThyORI control cells.

Given that FOXE1 has been described as a susceptibility gene in thyroid cancer, we first compared its level of expression in a panel of human thyroid cancer cell lines with its expression in the normal (immortalized) thyroid follicular cell line NThy-ori-3-1 (NThyOri). The cell lines used covered the spectrum of thyroid neoplasms, from well-differentiated PTC and follicular thyroid carcinomas (FTC) to anaplastic (undifferentiated) thyroid carcinoma (ATC), which is the most aggressive malignancy. Although there were some exceptions, overall *FOXE1* mRNA levels were significantly higher in control and in PTC-derived cells than in FTC-derived cells, with the lowest expression observed in ATC-derived cell lines (Fig. 1A and B). Western blotting analysis of FOXE1 protein confirmed the RNA expression data, showing significantly higher expression in PTC cells than in FTC and ATC cells, with some ATC lines having almost undetectable levels of FOXE1 (Fig. 1C). These results indicate that FOXE1 expression positively correlates with the differentiation status according to the histological sub-type origin of cell lines.

**Figure 2:**
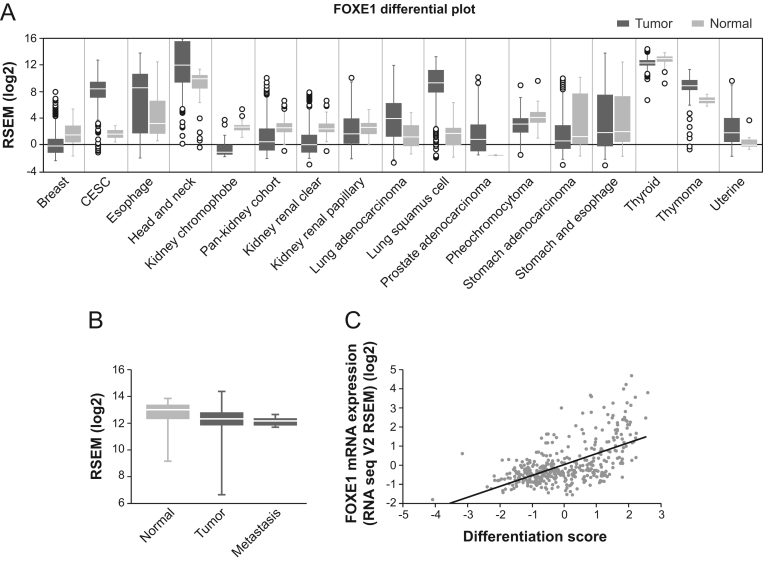
*FOXE1* expression in human tumors. (A) *FOXE1* relative mRNA expression in the indicated cancer types (CESC, cervical squamous cell carcinoma) obtained by Firebrowse analysis of TCGA database. (B) Box plot of *FOXE1* mRNA expression levels in thyroid normal tissue, papillary thyroid carcinoma (PTC), and metastases: data were obtained from TCGA database. (C) Correlation between *FOXE1* mRNA expression levels in thyroid tumors and differentiation score. Data were obtained from TCGA database (Pearson *r* = 0.5938).

To extend these observations to human tumors in general and in particular to thyroid tumors, we analyzed the pattern of *FOXE1* expression in a range of tumor samples and normal tissues using data acquired from The Cancer Genome Atlas (TCGA) (The Cancer Genome Atlas 2014) using the FireBrowse and Morpheus tools. We observed that *FOXE1* levels were elevated in several cancer types, with the most striking changes seen in esophagus and lung squamous cell carcinomas (Fig. 2A). By contrast, the expression levels of *FOXE1* in PTC and normal thyroid tissue were very similar (normal tissue *n* = 59, tumor samples *n* = 501) (Fig. 2B). Of note, the levels of FOXE1 in thyroid metastases (*n* = 8) were similar to those of the primary tumor samples and normal tissue (Fig. 2B), suggesting a role for FOXE1 in tumor progression. To further explore the implications of FOXE1 in thyroid cancer, we examined for correlations with aggressive clinical features. TCGA data from the public databases cBioportal and Cancer Regulome (Cerami *et al.* 2012, Gao *et al.* 2013) indicated that *FOXE1* levels did not correlate with the risk of recurrence or with extrathyroidal extension (Supplementary Fig. 1A). However, we observed a clear correlation between *FOXE1* expression and the differentiation state in PTC tumors (Fig. 2C), which is consistent with the data acquired in the thyroid tumor cells lines. This was also confirmed after analyzing *FOXE1* expression levels in a genome array of human tumors that encompass all histological variants (data not shown) (Oncomine Data Set (www.oncomine.org)) (Giordano *et al.* 2006). In addition, microarray studies have shown that *FOXE1* is among the 15 most downregulated genes in ATC (Tomas *et al.* 2012, von Roemeling *et al.* 2015) (Supplementary Fig. 1B).

### Genotyping of cancer risk FOXE1 SNPs rs965513 rs1867277 and the FOXE1 poly-alanine repeat region (rs71369530) in thyroid cancer cell lines

Association studies have identified two SNPs that are clearly linked to PTC and FTC in multiple populations (Wang *et al.* 2018). Both rs1867277 and rs965513 SNPs are located in the same disequilibrium block; that is, their alleles co-occur on the same haplotype more often than is expected by chance and are located 238 bp and 57 kb upstream of the *FOXE1* transcription start site, respectively (Gudmundsson *et al.* 2009, Landa *et al.* 2009). In addition, an association has been found between the SNP genotypes and expression levels of *FOXE1*. Thus, it was reported that in unaffected thyroid tissue, *FOXE1* expression was significantly lower in patients with the rs965513 AA genotype when compared with those with the GG genotype, but there was no significant correlation in PTC tumors (He *et al.* 2015*a*). Furthermore, the polymorphism in the length of the poly-A tract of *FOXE1* (rs71369530), which has been associated with thyroid cancer (Kallel *et al.* 2010), has been demonstrated to be in tight linkage disequilibrium with rs1867277, in addition to being associated (*FOXE1 ^16Ala^*) with PTC (Bullock *et al.* 2012).

**Figure 3:**
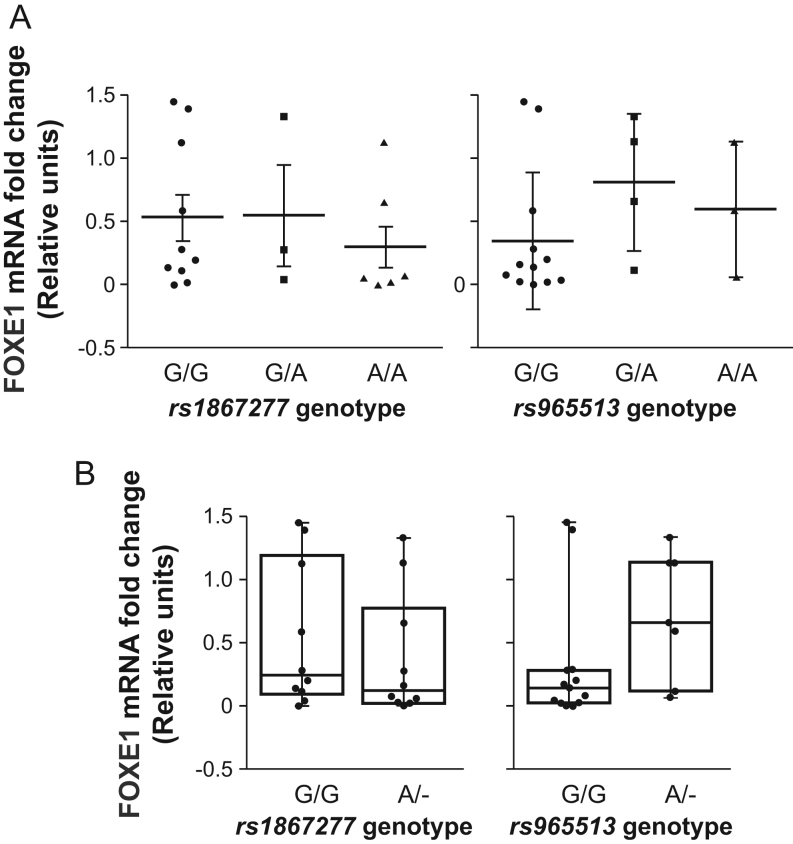
No correlation between single nucleotide polymorphism genotypes and *FOXE1* expression levels. (A) Representation of correlation of *FOXE1* mRNA expression and genotypes (GG, AG, or AA) of rs1867277 and rs965513. Values represent mean ± s.e.m. (B) Box plot of *FOXE1* mRNA expression in thyroid cancer cell lines *vs* genotypes with or without risk allele A of rs1867277 and rs965513. Values represent mean ± max to min.

To ascertain whether a correlation exists between SNP haplotypes and *FOXE1* expression in cancer, rs1867277, rs965513, and rs71369530 were genotyped by direct automated sequencing in the panel of human thyroid cancer cell lines. Allele variability was found in the analyzed cell lines, reflecting common variability in the general population, with minor allele frequency [A] of 0.5 for rs1867277 and 0.4 for rs965513 (Table 1). No significant correlation between the rs1867277 and rs965513 genotypes and the expression levels of *FOXE1* were observed in the thyroid cancer cell lines analyzed (Fig. 3A). Moreover, we failed to find a correlation between *FOXE1* expression and the G (G/G) or A (G/A or A/A genotype) allele (Fig. 3B). Finally, we confirmed that the most frequent *FOXE1* poly-A variant in our panel of thyroid cancer cells is *FOXE1 ^14Ala ^*followed by *FOXE1 ^16Ala^*. Other repeat lengths were rare in the thyroid cancer cell lines (Table 1). In addition, we failed to observe any correlation between the *FOXE1* poly-A variant and the expression levels of *FOXE1* in the thyroid cancer cell lines analyzed (data not shown).

**Table 1:** Genotypes of FOXE1 SNPs in thyroid cancer cell lines.

Cancer subtype	Cell line	Mutation	SNPs genotype			Other described mutations
			*rs1867277*	*rs965513*	*Poly Alanine*	
PTC	BCPAP	BRAF V600E	G/G	A/A	14/14	p53 D259Y/AKT1 copy gain
PTC	K1	BRAF V600E	A/A	A/A	16/16	PI3KCA E542K
PTC	KTC1	BRAF V600E	G/A	G/A	14/14	–
PTC	NIM	BRAF V600E	G/G	G/A	14/14	–
PTC	TPC1	RET/PTC1	G/G	G/G	14/14	–
FTC	FTC-133	PTEN R130*	A/A	A/A	16/16	p53 R273H
FTC	ML-1	–	G/G	G/G	14/14	p53 frameshift
FTC	TT206-C09	NRAS	G/A	G/G	14/14	p53 R273C
FTC	WRO	BRAF V600E	A/A	G/A	14/16	–
ATC	8505c	BRAF V600E	G/G	G/G	14/14	p53 R248G
ATC	C643	HRAS	A/A	G/G	14/14	p53 R248Q and K286E
ATC	Cal62	KRAS	G/G	G/G	14/14	p53 A161D
ATC	HTH7	NRAS	A/A	G/G	14/16	p53 G245S
ATC	HTH83	HRAS	G/A	G/G	12/14	–
ATC	KTC2	BRAF V600E	G/G	G/G	14/14	–
ATC	OCUT2	BRAF V600E	G/G	G/G	14/14	PI3KCA H1047R
ATC	SW1736	BRAF V600E	A/A	G/G	14/16	p53 Null
ATC	T235	BRAF V600E	G/G	G/G	14/14	–
ATC	T238	BRAF V600E	A/A	G/G	16/16	p53 S183*/PI3KCA E542K
–	NThyORI	–	G/G	G/A	14/14	–

The genotypes of the rs1867277, rs965513 SNPs and the length of the poly-alanine tract are shown in the central columns. The cancer subtype from which the cell lines originated, as well as the driver mutations, is also shown.

These data suggest that FOXE1 expression in tumoral thyroid cell lines is independent of the allele/genotype.

### Analysis of migration in thyroid cancer cell lines

**Figure 4:**
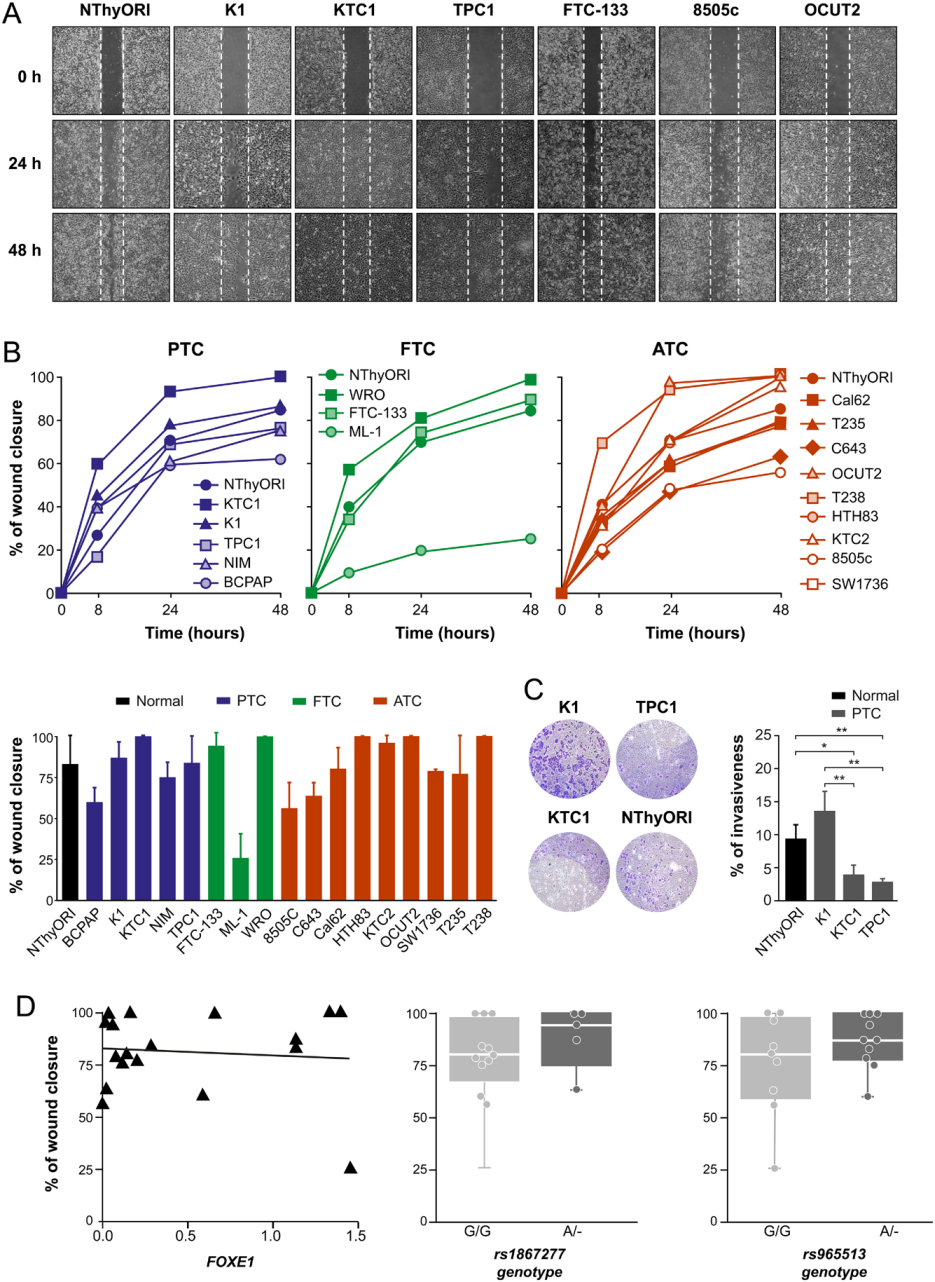
Analysis of thyroid cancer cell migration and invasion. (A) Representative images of wound-healing assay of PTC cell lines: K1, KTC1, and TPC1; FTC cell line: FTC-133; ATC cell lines: 8505c, OCUT2 and NThyORI cells. (B) Time course of wound closure in PTC-, FTC-, and ATC-derived cells. Cells were photographed at 0, 8, 24 and 48 h, and wound closure area was quantified using ImageJ software. Bar graph shows wound closure after 48 h of migration. Values represent mean ± s.e.m. of the percentage of the closure of original wound from three independent experiments performed in triplicate. (C) NThyORI, K1, KTC1, and TPC1 cells were seeded in the upper chambers of Transwells, allowed to migrate for 18 h, and photographed. Left: representative images of the lower chamber (invading cells). Right: percentage of invasiveness by direct measurement with ImageJ. Values represent mean ± s.e.m. from three independent experiments performed in triplicate. *P < 0.05, **P < 0.01. (D) Correlation analysis between migration, SNP genotype, and FOXE1 expression levels. Left: representation of correlation between wound closure and levels of FOXE1 (Pearson *r* = −0.091). Center and Right: representation of the correlation between wound closure and genotype in rs1867277 and rs965513 (G/G; –/A).

In an attempt to discern the role of *FOXE1* in thyroid tumorigenesis, and considering that this gene is involved in the migration of thyroid cells from the pharyngeal floor to the trachea during development (De Felice *et al.* 1998), we hypothesized that FOXE1 could be involved in thyroid tumor cell migration. To study intrinsic cellular motility, we first performed scratch wound-healing assays in thyroid cancer cell lines. Results showed that among the PTC cell lines, K1 and KTC1 showed the highest cellular migration, which was greater than that of control NThy-ori-3-1 cells (Fig. 4A and B). Likewise, the FTC cell lines WRO and FTC-133 and the ATC cell lines T238 and OCUT2 showed the greatest extent of wound closure after 24 and 48 h (Fig. 4B). By contrast, the 8505c and C643 cell lines exhibited the lowest percentages of wound closure among ATC-derived cells (Fig. 4A and B).

We also performed Matrigel assays to evaluate the invasive capacity of the three PTC cell lines showing the greatest migration capacity compared with NThy-ori-3-1 cells. We found that K1 cells had a significantly higher invasive capacity through Matrigel than KTC1, TPC1, and NThy-ori-3-1 cells (Fig. 4C). In turn, KTC1 and TPC1 cells had a significantly lower invasive capacity than control NThy-ori-3-1 cells (Fig. 4C). We then evaluated whether a correlation exists between SNP genotype, FOXE1 levels, and cellular migration. We found that the accelerated wound closure did not correlate with expression levels of FOXE1 (Pearson *r* = −0.091) or with the presence of one copy of the [A] allele in rs1867277 and rs965513 (Fig. 4D).

### FOXE1 regulates genes involved in epithelial-to-mesenchymal transition

**Figure 5:**
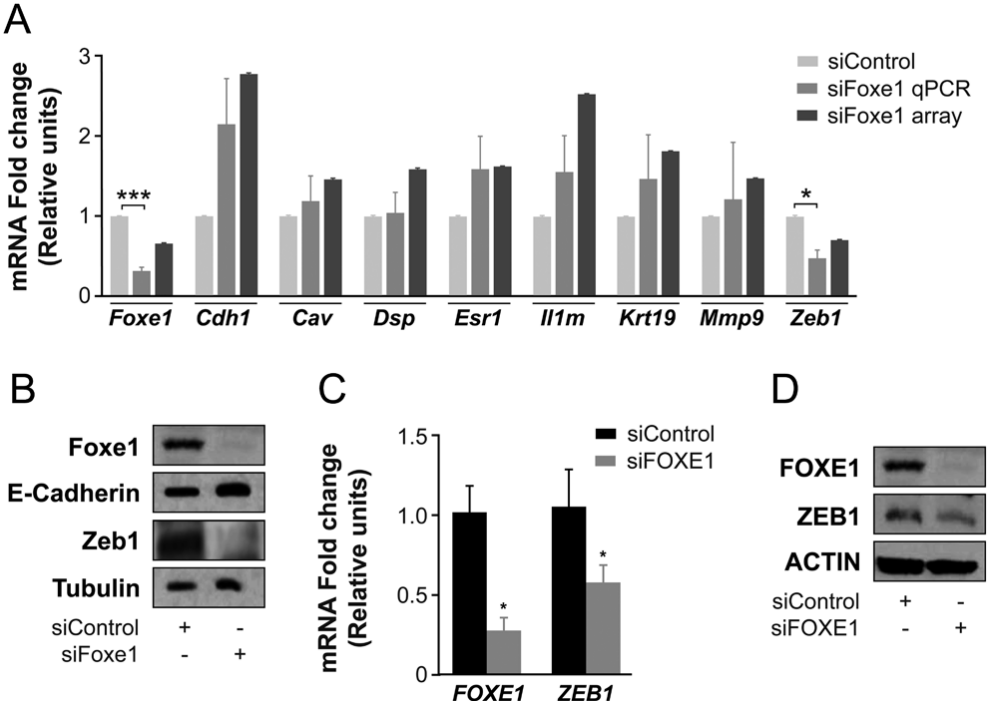
Experimental validation of microarray results by quantitative PCR. (A) Relative expression of seven EMT-specific genes in *Foxe1*-silenced PCCl3 cells assessed by qPCR. Silenced *Foxe1* microarray data are from Fernandez *et al*. (2013). As controls, we evaluated *Foxe1* and *Cdh1* mRNA expression levels. Relative gene expression in silenced *Foxe1* (si*Foxe1*) samples was calculated using the corresponding siScramble samples as a reference. Values represent mean ± s.e.m. of four independent experiments. *P < 0.05, ***P < 0.001 *vs* siScramble. (B) Total protein extracts from PCC13 cells were analyzed to assess the protein levels of Foxe1, E-Cadherin, and Zeb1; tubulin was used as loading control. A representative Western blot of four independent experiments is shown. (C) *FOXE1* and *ZEB1* expression in the K1 thyroid cancer cell line after 48 h of siFOXE1 transfection assessed by qPCR analysis using the corresponding siScramble samples as a reference. Values are mean ± s.e.m. of three independent experiments. *P < 0.01 *vs* siScramble. (D) Total protein extracts were analyzed to assess the protein levels of FOXE1 and ZEB1 after 72 h of siFOXE1 transfection of K1 cells. Actin was used as a loading control. A representative Western blot analysis of four independent experiments is shown.

In a previous genome-wide screening analysis in *Foxe1-*silenced rat PCCl3 cells, we identified several novel genes potentially regulated by Foxe1 (Fernandez *et al.* 2013). Among the upregulated genes was *Cdh1*, which is widely involved in EMT (Peinado *et al.* 2004, Lamouille *et al.* 2014). However, we were unable to demonstrate direct binding of Foxe1 to the *Cdh1* promoter (Fernandez *et al.* 2013). Using this previous analysis as a guide, and to assess the possible role of FOXE1 in regulating invasion and migration, we analyzed the mRNA levels of seven randomly chosen EMT-associated genes (*Cav*, *Dsp*, *Esr1*, *Il1rn*, *Krt19*, *Mmp9,* and *Zeb1*) from the list of putative *Foxe1* targets (Fernandez *et al.* 2013). We first silenced *Foxe1* in PCCl3 cells and confirmed that the mRNA level of *Foxe1* was decreased and the mRNA level of *Cdh1* was increased as compared with control-silenced cells (Fig. 5A). Expression qPCR analysis of the seven genes showed that six genes were moderately upregulated and one (*Zeb1*) was significantly downregulated in *Foxe1-*silenced PCC13 cells (Fig. 5A), which is in accord with our previous expression array analysis (Fernandez *et al.* 2013). As expected, Western blotting showed that protein levels of Zeb1 were lower in *Foxe1*-silenced cells than in control-silenced cells, whereas the opposite was observed for E-Cdh1 (Fig. 5B). Although on the surface these results might seem contradictory considering that *Foxe1* is a differentiation-related gene, they can be reconciled if *Foxe1* has a *bona fide* role in migration and consequently in EMT. To test whether FOXE1 regulates ZEB1 abundance in human thyroid cancer cells, we silenced its expression in K1 cells, which exhibited the greatest migration and invasion ability of all the PTC lines analyzed. In accord with the findings in rat PCCI3 cells, FOXE1 silencing resulted in a significant decrease in *ZEB1* mRNA (Fig. 5C) and protein (Fig. 5D) abundance, suggesting that ZEB1 is a target of FOXE1.

### FOXE1 binds specifically to the *ZEB1* promoter and regulates its transcriptional activity

**Figure 6:**
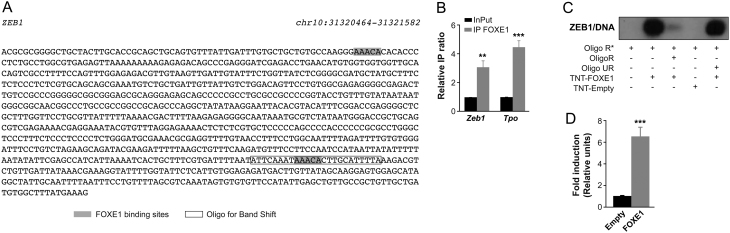
FOXE1 binding to and transcriptional activation of the human *ZEB1* promoter. (A) Chromosomal location of putative FOXE1-binding sites in the human *ZEB1* promoter. We searched for FOXE1-binding sites in promoter regions (−1010/+26 bp relative to the transcription start site) of *ZEB1*. FASTA promoter sequence of *ZEB1* was extracted from the Ensemble database (http://www.ensemble.org). (B) ChIP experiments for FoxE1 binding to *Zeb1* promoter and qPCR analysis of immunoprecipitated chromatin in PCCl3 cells using a Foxe1 antibody. The enrichment of target sequence was calculated as the immunoprecipitation ratio (arbitrary units) relative to the negative control *Cis* 2.5 kb and normalized to the relative amplification in the input sample. A sequence from the *Tpo* promoter was used as a positive control. Values represent mean ± s.e.m. of four independent experiments each performed in triplicate. (C) EMSA assays were performed with a ^32^P-labeled probe containing the specific recognition sequence for FOXE1. The ^32^P-labeled probe (Oligo R*) was incubated alone (lane 1), with TNT-translated FOXE1 (lanes 2, 3, and 5) or with TNT-translated proteins from an empty vector (lane 4). Competition was performed with an excess of unlabeled related (Oligo R, lane 3) or unrelated oligonucleotides (Oligo UR, lane 5). (D) The human *ZEB1* promoter was cotransfected into HeLa cells with 3 µg of the empty expression vector or with 3 µg of a vector harboring the cDNA for FOXE1. *ZEB1* promoter activity is expressed as fold-induction relative to the activity observed with the empty expression vector. Luciferase activity was normalized to *Renilla* activity derived from the cotransfected pRL-SV40 to adjust for transfection efficiency. Values represent mean ± s.d. of four independent experiments. ***P < 0.001 *vs* control.

Given the aforementioned results, we next searched for canonical FOXE1-binding sites within the proximal human *ZEB1* promoter (±1010 bp), finding two potential FOXE1-binding domains (Fig. 6A). We performed ChIP analysis to determine whether Foxe1 binds to the *Zeb1* promoter sequence in PCCl3 cells. We used a polyclonal antibody against Foxe1 and analyzed the immunoprecipitated DNA of two independent experiments using qPCR. Foxe1 binding to the *Tpo* promoter was used as a positive control (Fernandez *et al.* 2013). ChIP analysis showed unequivocally that Foxe1 interacts with the *Zeb1* promoter when normalized to the control (Fig. 6B). Also, an electrophoretic mobility shift assay showed that recombinant FOXE1 binds specifically to the probe derived from the ZEB1 promoter (Fig. 6C).

Lastly, we cotransfected HeLa cells with a FOXE1 expression vector and a luciferase reporter vector containing the *ZEB1* promoter. Reassuringly, results showed a significant six-fold increase in *ZEB1* promoter activity by FOXE1 expression (Fig. 6D). Overall, these results indicate that FOXE1 functionally transactivates the *ZEB1* promoter.

### FOXE1 promotes thyroid cancer cell migration and invasion

**Figure 7:**
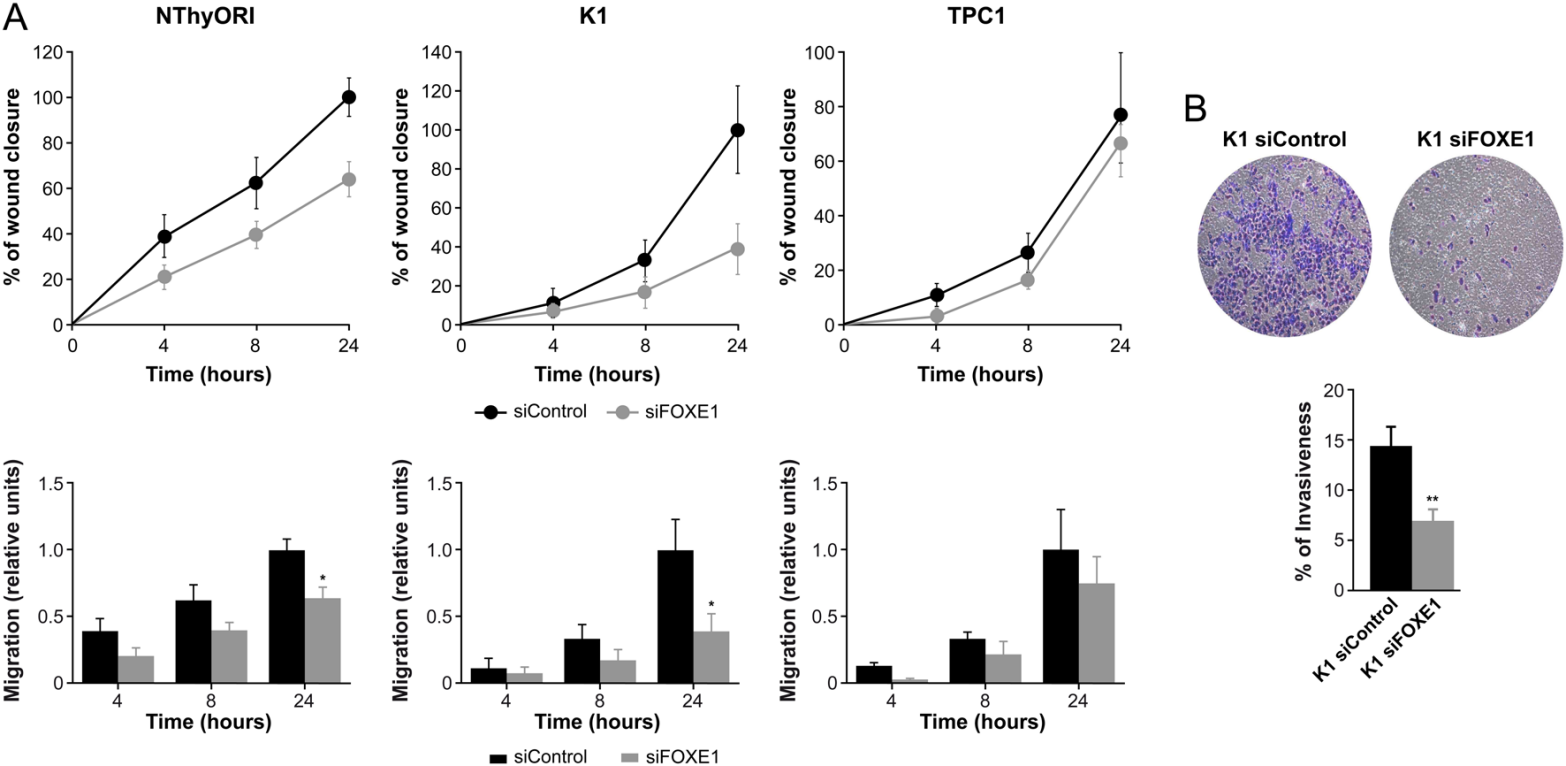
FOXE1 modulates migration and invasion in thyroid cancer cells. (A) Time course of wound closure in NThyORI, K1, and TPC1 cells silenced or not for FOXE1 expression (upper panel). Cells were photographed at 0, 4, 8, and 24 h, and wound closure area was quantified using ImageJ software. Quantification of migration rates in FOXE1-silenced cells *vs* control cells are shown in lower panel. Bar graph shows migration after 4, 8, and 24 h. (B) Invasiveness of K1 cells after FOXE1 silencing. Top: representative images of the lower chamber (invading cells). Bottom: percentage of invasiveness relative to siControl cells. Values represent mean ± s.e.m. from three independent experiments *P < 0.05, **P < 0.01. A full color version of this figure is available at https://doi.org/10.1530/ERC-19-0156.

We investigated whether FOXE1 affects the migration and invasion capability of thyroid cancer cells. We thus silenced *FOXE1* expression in NThy-ori-3-1, K1, and TPC1 cells and repeated the scratch-wound and Transwell analysis. The results showed that *FOXE1* silencing significantly impaired cell migration relative to control non-silenced cells (Fig. 7A). In addition, FOXE1 depletion significantly suppressed the invasion of K1 thyroid cancer cells in Transwell assays with Matrigel (Fig. 7B). Reciprocal experiments in NThy-ori-3-1, K1, and TPC1 cells showed that the over-expression of FOXE1 markedly increased, in a time-dependent manner, cell migration ability (Supplementary Fig. 2). These findings indicate that FOXE1 is closely associated with metastatic phenotypes of thyroid cancer cells.

### ZEB1 knockdown suppresses cell migration and invasion in thyroid cancer cells

**Figure 8:**
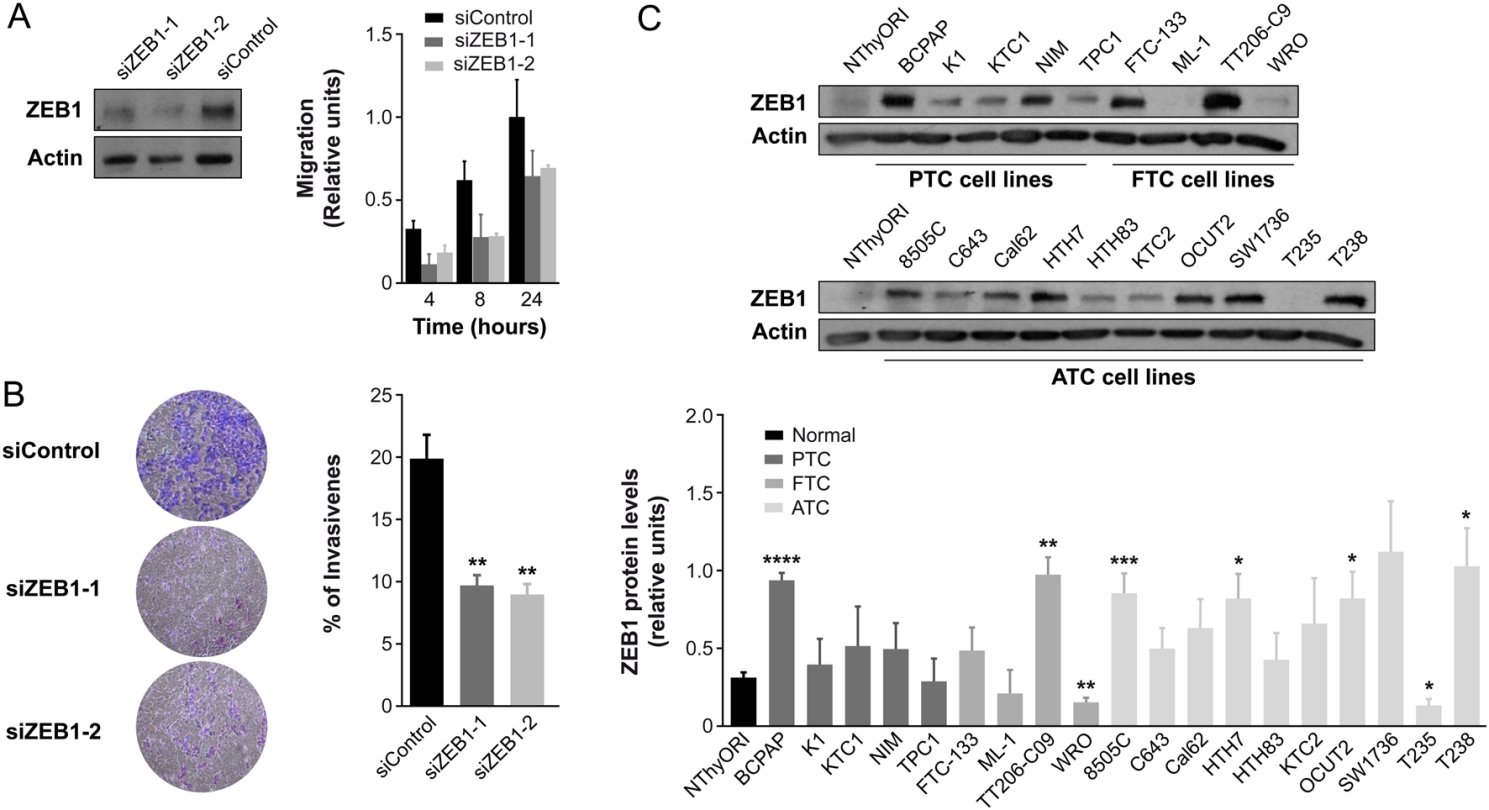
ZEB1 silencing reduces migration and invasion in thyroid cancer cells. (A) Analysis of *ZEB1* silencing in K1 cells (left panel). Total protein extracts were analyzed by Western blot to assess the protein levels of ZEB1 after 48 h of siZEB1-1 and siZEB1-2 transfection. Actin was used as a loading control. A representative Western blot analysis of three independent experiments is shown. Quantification of migration rates in ZEB1-silenced cells *vs* control cells is shown in the right panel. (B) Invasion ability of ZEB1-silenced K1 cells. Left: representative images of the lower chamber (invading cells). Right: percentage of invasiveness relative to siControl cells. Values represent mean ± s.e.m. from four independent experiments **P < 0.01. (C) Upper panel: Representative Western blot of three independent experiments showing ZEB1 protein levels in thyroid cell lines. Actin was used as a loading control. Lower panel: ZEB1 protein expression in thyroid cell lines normalized to expression of actin. Values represent mean ± s.e.m. of three independent experiments. *P < 0.05, **P < 0.001, ****P < 0.0001 *vs* NThyORI control cells. A full color version of this figure is available at https://doi.org/10.1530/ERC-19-0156.

Given the potentially crucial role of ZEB1 in EMT, we hypothesized that FOXE1 may regulate the EMT process in thyroid cancer cells, at least partly, by targeting ZEB1. Thus, we examined the effects of *ZEB1* silencing on cell migration and invasion. Wound healing and Transwell analysis showed that ZEB1 is likely important for the migration and invasion of thyroid cancer cells, as both parameters were decreased in silenced cells (Fig. 8A and B). We also examined the expression of ZEB1 in a panel of human thyroid cancer cell lines, but we could not establish a clear correlation between ZEB1 and FOXE1 expression (Fig. 8C).

## Discussion

Beyond its role in organogenesis and differentiation (De Felice & Di Lauro 2004, Fernandez *et al.* 2015), FOXE1 has been described as being strongly associated with susceptibility to several types of cancer (Brune *et al.* 2008, Venza *et al.* 2010, Park *et al.* 2012) including PTC (Gudmundsson *et al.* 2009, Landa *et al.* 2009, Bullock *et al.* 2012, He *et al.* 2015*a*). These seemingly opposing roles are intriguing and have been the focus of our study. Indeed, several genes have been identified with dual roles as oncogenes and tumor suppressors (Shen *et al.* 2018), but few examples exist of genes related to differentiation, and therefore necessary for normal cell development, and at the same time involved in cancer. This characteristic, however, has been attributed to the Forkhead-box family of transcription factors, as they are involved in differentiation, embryogenesis, longevity, DNA repair, and carcinogenesis (Katoh *et al.* 2013). Accordingly, FOXE1 might also exert this dual action.

The role of Foxe1 in development and differentiation is well understood (Santisteban *et al.* 1992, Aza-Blanc *et al.* 1993, Ortiz *et al.* 1997, 1999, De Felice *et al.* 1998, De Felice & Di Lauro 2004, Fernandez *et al.* 2015, Lopez-Marquez *et al.* 2019), yet little is known about its potential role in thyroid carcinogenesis, or how different allelic variants in or near *FOXE1* are associated with thyroid cancer risk. In the present study, we characterized FOXE1 expression levels in a panel of thyroid cancer cell lines and analyzed the potential role of FOXE1 in the regulation of EMT.

FOXE1 expression levels are unaltered or are upregulated in human tumors, suggesting that it might be important in the initiation and progression of these tumors (Sato *et al.* 2003, Eichberger *et al.* 2004, Venza *et al.* 2010, Park *et al.* 2012, Sugimachi *et al.* 2016). Loss of thyroid-specific proteins and differentiation markers is common in thyroid carcinogenesis. Indeed, several studies have reported the abnormal expression of thyroid-specific transcription factors in some thyroid carcinomas and propose that their deregulation is a pivotal event for the initiation and progression of thyroid neoplasms (Fabbro *et al.* 1994, Ros *et al.* 1999, Zhang *et al.* 2006). In the case of FOXE1, it has been described that nuclear expression is lost according to the degree of tumor dedifferentiation, which seems to be related to the progression of thyroid tumorigenesis (Zhang *et al.* 2006, Bychkov *et al.* 2013). Conversely, it has also been described that FOXE1 is overexpressed in PTC, and shows an aberrant cytoplasmic location in PTC cells, which again has been related to cancer cell biology (Nonaka *et al.* 2008, Bychkov *et al.* 2013). These two seemingly opposing processes makes the study of this factor in thyroid cancer challenging.

Our analysis of FOXE1 expression in a panel of thyroid cancer cell lines shows that FOXE1 levels inversely correlate with differentiation degree according to histological sub-type origin of cell lines, which is consistent with data on the expression patterns of *FOXE1* in PTC and normal tissues by TCGA and fits with data of *FOXE1* expression in a genome array of human tumors that include all histological variants (Giordano *et al.* 2006). It should be mentioned that other recent studies have reported overexpression of FOXE1 in PTC samples as well as in K1 and TPC1 cell lines (Ding *et al.* 2019, Ma *et al.* 2019), which contrasts with our data. We do not have an explanation for this apparent discrepancy other than the use of different antibodies in the studies; however, our results showing lower levels of FOXE1 in PTC cells than in control cells are in agreement with data from TCGA on more than 500 samples of patients with PTC.

We genotyped the rs1867277 and rs965513 SNPs in the same panel of thyroid cancer cell lines, as different variants in and near *FOXE1* have been associated with the predisposition to PTC (Gudmundsson *et al.* 2009, Landa *et al.* 2009, Matsuse *et al.* 2011). In this regard, He *et al*. reported that the risk [A] allele of DNA variant rs965513 was associated with low expression levels of *FOXE1* in unaffected thyroid tissue, but no correlation was found between rs965513 genotype and *FOXE1* levels in PTC tissues (He *et al.* 2015*a*). Further, in a functional study of rs1867277, Landa *et al*. revealed that the risk [A] allele led to the differential recruitment of USF1 and USF2 transcription factors, affecting the transcriptional regulation of *FOXE1* and conferring a role in the pathogenesis of PTC. In our correlation studies, however, we failed to observe an association between *FOXE1* levels and the risk [A] allele of rs965513 or of rs1867277 in the thyroid cancer cell lines analyzed. A possible explanation for this is the complexity of the 9q22 region, as there is evidence supporting a different regulatory model that may govern *FOXE1* promoter activity (He *et al.* 2015*b*, Wang *et al.* 2017), together with possible epigenetic modifications due to the proximity of CpG islands (Abu-Khudir *et al.* 2014) and the potential involvement of other transcription factors (Landa *et al.* 2009, Lopez-Marquez *et al.* 2019). Similarly, although the existence of a relationship between the poly-A tract and PTC tumors has been described (Bullock *et al.* 2012), we did not observe this in the panel of cell lines analyzed herein.

FOXE1 plays a crucial role in thyroid morphogenesis by promoting thyroid precursor cell migration during gland development (De Felice *et al.* 1998), suggesting the involvement of FOXE1 in cell migration and EMT. We therefore analyzed cell migration in the panel of thyroid cancer cell lines in relation to FOXE1 expression and SNP genotype. Although FOXE1 levels positively correlated with migration rate in some cell lines, we could not establish a correlation between FOXE1 expression and the ability of thyroid cancer cell lines to migrate and invade. Nevertheless, silencing of FOXE1 expression resulted in impaired thyroid cancer cell migration and invasion, and the opposite was observed after FOXE1 overexpression. Similarly, the presence of one copy of the [A] allele in rs1867277 and rs965513 did not significantly correlate with accelerated wound closure; however, it seems that there is a high migration capacity in cell lines containing the [A] risk allele of SNP rs1867277.

Foxe1 binds to DNA sequences present in the promoters of thyroglobulin (Santisteban *et al.* 1992) and thyroperoxidase (Aza-Blanc *et al.* 1993), promoting their transcriptional activation. In a previous study, we identified novel Foxe1 downstream targets using expression arrays in *Foxe1*-silenced thyroid epithelial cells (Fernandez *et al.* 2013), supporting the involvement of FOXE1 in relevant biological processes and pathways. One of the hallmarks of EMT is the functional loss of E-cadherin (encoded by *Cdh1*), which was upregulated in *Foxe1*-silenced thyroid cells, suggesting that FOXE1 modulates the expression of *Cdh1*. In this study, we show that ZEB1, a key factor that modulates E-cadherin expression and the induction of EMT, is regulated by FOXE1 in thyroid cells. In addition, we demonstrate a direct interaction of FOXE1 with the *ZEB1* promoter and an increase in ZEB1 transcriptional activity in FOXE1-transfected cells. Interestingly, loss-of-function experiments revealed that cells silenced for ZEB1 show blunted migration and invasion relative to control non-silenced cells, a behavior similar to that observed in silenced FOXE1 cells, which clearly demonstrates that FOXE1 regulates migration and invasion in thyroid cancer cells, at least in part, through ZEB1.

Taken all this together, we postulate that FOXE1 has a crucial role in thyroid tumor cell migration and invasion, as shown by the results of loss/gain-of-function of FOXE1 on migration/invasion, and with the increasing evidence of the role of forkhead box proteins in the development and progression of cancer (Myatt & Lam 2007, Katoh *et al.* 2013).

Our results are also consistent with other studies, demonstrating that FOXE1 can interact with other factors and transactivate key genes in cancer such as *SNAIL* (Xu *et al*. 2015), an E-cadherin transcriptional repressor, or *TERT* (telomerase reverse transcriptase), which is coregulated by FOXE1 and the ETS factor ELK1 (Bullock *et al.* 2016). Along this line, it would be interesting to search for FOXE1 interacting partners in thyroid cancer, which may reveal unique or separate signaling pathways.

In conclusion, we have identified ZEB1 as a *bona fide* target of FOXE1 in thyroid cancer cells, which provides new insights into the role of FOXE1 in regulating EMT in thyroid cancer.

## Supplementary Material

Supplementary Figure 1. Correlation between FOXE1 mRNA levels and high-, intermediate- or low-risk (A) or extrathyroid extension (B). TCGA datasets were analyzed with cBioPortal. (C) Analysis of GEO datasets of microarray assays of FOXE1 expression in ATC of two studies: Tomas et al. 2012 (1) and von Roemeling et al. 2015 (2). Upper table shows the probe ID used and respective P-values. Lower bar graphs represent expression levels of FOXE1 in ATC vs. normal thyroid controls, respectively. ****P<0.0001 vs. control.

Supplementary Figure 2. FOXE1 modulates migration in thyroid cancer cells. Time course of wound closure in NThyORI, K1 and TPC1 cells transfected with FOXE1 or an empty vector (upper panel). Cells were photographed at 0, 4, 8 and 24 hours and wound closure area was quantified using ImageJ software. Quantification of migration rates in FOXE1-transfected cells vs. control cells are shown in lower panel. Bar graph shows migration after 4, 8, and 24 h. Values represent mean ± SEM from three independent experiments *P< 0.05.

Supplementary Table 1. Primers used for determination on gene expression levels

Supplementary Table 2. Oligos used for SNPs genotyping

Supplementary Table 3. Oligos used for ChIP analysis

## Declaration of interest

The authors declare that there is no conflict of interest that could be perceived as prejudicing the impartiality of the research reported.

## Funding

This work was supported by grants SAF2016-75531-R from Ministerio de Ciencia, Innovación y Universidades (MICIU), Spain, Fondo Europeo de Desarrollo Regional FEDER, B2017/BMD-3724 from Comunidad de Madrid, and GCB14142311CRES from Fundación Española Contra el Cáncer (AECC).
