# Supplementary figures and images for: FOXE1 regulates migration and invasion in thyroid cancer cells and targets ZEB1
Source: Endocr Relat Cancer. 2019 Dec 16;27(3):137–51. doi: 10.1530/ERC-19-0156 (PMC6993207; doi:10.1530/ERC-19-0156)

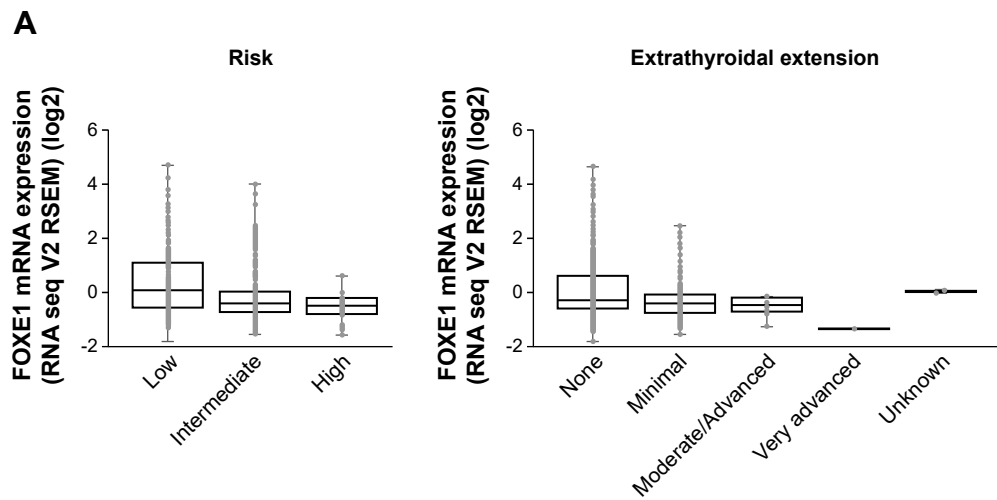

**B**

| Study | ID        | adj.P.Val | P.Value  | t     | B    | logFC |
|-------|-----------|-----------|----------|-------|------|-------|
| 1     | 206912_at | 1.39E-19  | 5.71E-22 | -15.4 | 39.8 | -5.43 |
| 2     | 206912_at | 3.08E-06  | 2.46E-08 | -7.9  | 9.26 | -5.38 |

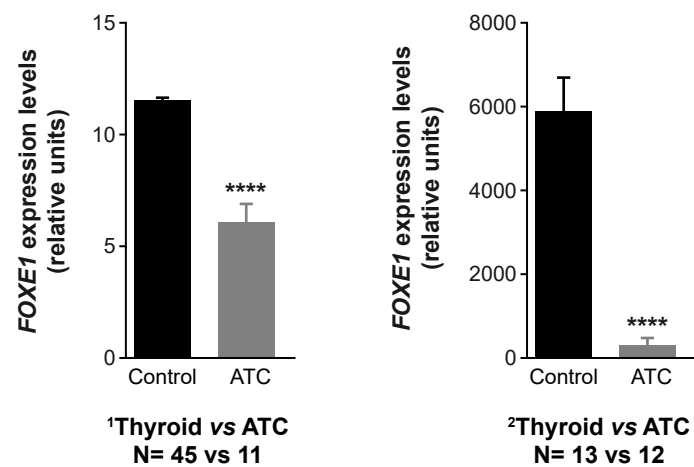

Supplement: Supplementary Figure 1. Correlation between FOXE1 mRNA levels and high-, intermediate- or low-risk (A) or extrathyroid extension (B). TCGA datasets were analyzed with cBioPortal. (C) Analysis of GEO datasets of microarray assays of FOXE1 expression in ATC of two studies: Tomas et al. 2012 (1) and vo [file supplementary_figure_1.pdf]

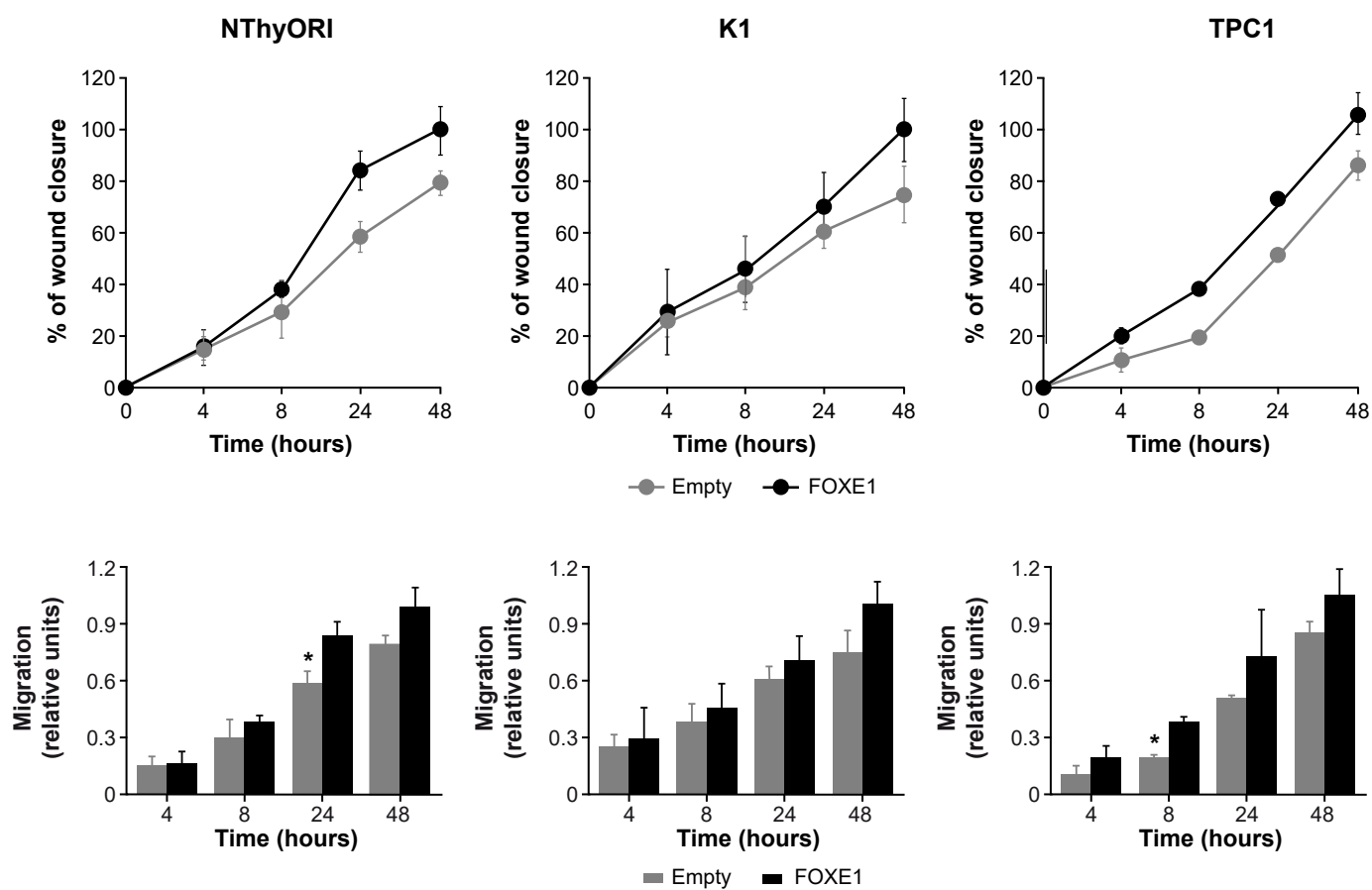

Figure Supp. 2

Supplement: Supplementary Figure 2. FOXE1 modulates migration in thyroid cancer cells. Time course of wound closure in NThyORI, K1 and TPC1 cells transfected with FOXE1 or an empty vector (upper panel). Cells were photographed at 0, 4, 8 and 24 hours and wound closure area was quantified using ImageJ software.  [file supplementary_figure_2.pdf]
