# Supplementary material for: FOXE1 regulates migration and invasion in thyroid cancer cells and targets ZEB1
Source: Endocr Relat Cancer. 2019 Dec 16;27(3):137–51. doi: 10.1530/ERC-19-0156 (PMC6993207; doi:10.1530/ERC-19-0156)
Supplement: Supplementary Table 1. Primers used for determination on gene expression levels [file supplementary_table_1.pdf]

Supplementary Table 1. Primers used for determination on gene expression levels

Gene	Specie	Oligonucleotide Sequence	
		Forward	Reverse
FoxE1	Rat	TCATCACCGAGCGCTTCCCGTT	GCGGCTGCATCGTGCATGTA
Cdh1	Rat	GTTTGCTCGGCGTTTGCCCG	ACAAAGCCACGAGGAGACCTGC
Cav	Rat	TTGGCCTTCATTGCGGGTAT	AGAGGAGAAGATGCGCCCTA
Dsp	Rat	AGCATCCAGCGTCAGACAAA	GGAGGGGCATGTTCTTGTCA
Esr1	Rat	GCGCAAGTGTTACGAAGTGG	GGTTGGCAGCTCTCATGTCT
Il1rn	Rat	AAGCTGTGCCTGTCTTGTGT	TCTCGGAGCGGATGAAGGTA
Krt19	Rat	GACGCGGTGGAAGTTTTAGTG	GCGGGCATTGTCGATCTGTA
Zeb1	Rat	GTGGATCCCCAGAGCGTTAC	ACCGCTGAAGCAAAAGAGGA
Mmp9	Rat	TGGGATGTACGCATGTGACC	TACAGATTCCGCACTCGTGG
Gus	Rat	CATGACGAACCAGTCACCAC	ACGGTCTGCTTCCCATACAC
GADPH	Human	GGTCTCCTCTGACTTCAACA	GTGAGGGTCTCTCTCTTCCT
FOXE1	Human	TGAGCCAGCGTAGGGACGAAAA	CCACCTCCTCCCGTTTACAGAGTA
ZEB1	Human	GCCAATAAGCAAACGATTCTG	TTTGGCTGGATCACTTTCAAG
CDH1	Human	CAGCACGTACACAGCCCTAA	ACCTGAGGCTTTGGATTCCT
