# Supplementary material for: FOXE1 regulates migration and invasion in thyroid cancer cells and targets ZEB1
Source: Endocr Relat Cancer. 2019 Dec 16;27(3):137–51. doi: 10.1530/ERC-19-0156 (PMC6993207; doi:10.1530/ERC-19-0156)
Supplement: Supplementary Table 2. Oligos used for SNPs genotyping [file supplementary_table_2.pdf]

Supplementary Table 2. Oligos used for SNPs genotyping

**Oligo Name**	**Orientation**	**Sequence**
rs965513	Forward	AATGTAGGTTTTTGGTGATGGTATGG
	Reverse	GTGAGAACAGACTAATACATCTTCTTTTTAATTT
rs1867277	Forward	CTTCAGCCGGAGACCAGAGT
	Reverse	CAGACAGAGGCTCGGGAGTG
Poly-A tract	Forward	CTCGGACCTCTCCACCTACC
	Reverse	GCGCGTAGCCTGCATAGAC
