# Supplementary material for: FOXE1 regulates migration and invasion in thyroid cancer cells and targets ZEB1
Source: Endocr Relat Cancer. 2019 Dec 16;27(3):137–51. doi: 10.1530/ERC-19-0156 (PMC6993207; doi:10.1530/ERC-19-0156)
Supplement: Supplementary Table 3. Oligos used for ChIP analysis [file supplementary_table_3.pdf]

Supplementary Table 3. Oligos used for ChIP analysis

**Oligo Name**	**Orientation**	**Sequence**
Zeb1	Forward	CTGGCAGCAGTCCTTTCCTT
	Reverse	TCCCTTGAACAATGCTGCCA
Tpo	Forward	AGCAAGGACACACAAGCACTT
	Reverse	CTCCACTGAAGAAGCAGGCTG
2 kb Cis	Forward	GAGGAGAGGGTTATGGCTGC
	Reverse	TCCCCTTGGCATTCAGTCAG
2.5 kb Cis	Forward	GACAGGAGGTGTCAGGTGTG
	Reverse	GAGCCCATTTCCCCTCAACA
